# Baicalin Magnesium Salt Attenuates Lipopolysaccharide-Induced Acute Lung Injury via Inhibiting of TLR4/NF-*κ*B Signaling Pathway

**DOI:** 10.1155/2021/6629531

**Published:** 2021-06-08

**Authors:** Lin Zhang, Lukun Yang, Xiaowei Xie, Hongyue Zheng, Hangsheng Zheng, Lizong Zhang, Cuizhe Liu, Ji-Gang Piao, Fanzhu Li

**Affiliations:** ^1^College of Pharmaceutical Sciences, Zhejiang Chinese Medical University, Hangzhou, Zhejiang 310053, China; ^2^Hebei Province Key Laboratory of Research and Development for Chinese Medicine, Institute of Traditional Chinese Medicine, Chengde Medical College, Chengde, Hebei 067000, China; ^3^Libraries of Zhejiang Chinese Medical University, Zhejiang Chinese Medical University, Hangzhou, Zhejiang 310053, China; ^4^Academy of Chinese Medical Sciences, Zhejiang Chinese Medical University, Hangzhou, Zhejiang 310053, China

## Abstract

Baicalin (BA) magnesium salt (BA-Mg) is a good water-soluble ingredient extracted from *Scutellaria baicalensis Georgi*, a commonly used traditional Chinese medicine. This study is aimed at investigating whether BA-Mg could exert a better protective effect on lipopolysaccharide- (LPS-) induced acute lung injury (ALI) in mice and illuminate the underlying mechanisms in vivo and in vitro. Mice were intraperitoneally administrated with equimolar BA-Mg, BA, and MgSO_4_ before LPS inducing ALI. Lung tissues and bronchoalveolar lavage fluid were collected for lung wet/dry ratio, histological examinations, cell counts, and biochemical analyses at 48 h post-LPS exposure. Meanwhile, the protein expressions of TLR4/NF-*κ*B signaling pathway and proinflammatory cytokines in lung tissues and lung bronchial epithelial cells (BEAS-2B) were detected. The results showed BA-Mg pronouncedly ameliorated LPS-induced inflammatory response and histopathological damages, elevated antioxidant enzyme activity (SOD), and downregulated myeloperoxidase (MPO) and malonaldehyde (MDA) levels through the inhibition of TLR4/NF-*κ*B signaling pathway activation. Moreover, the effect of BA-Mg was significantly better than that of BA and MgSO_4_ in ameliorating symptoms. Overall, BA-Mg can effectively relieve inflammatory response and oxidative stress triggered by LPS, indicating it may be a potential therapeutic candidate for treating ALI.

## 1. Introduction

Acute lung injury (ALI) is a life-threatening disorder that is mainly caused by direct and indirect factors, including sepsis, harmful inhalation, burn injury, and trauma [1, 2]. It is clinically shown as overwhelming pulmonary inflammation, widespread capillary leakage, acute onset, and progressive hypoxemia [3]. The currently major therapies include conservative fluid strategy, extracorporeal membrane oxygenation, and prone position ventilation [4]. Although advances in prognosis and treatment modalities, its morbidity and mortality are still high [5, 6]. At present, there are no effective medicines or measures to control ALI [7]. Consequently, it is an urgent need for seeking a novel effective drug for ALI.

**Figure 1:**
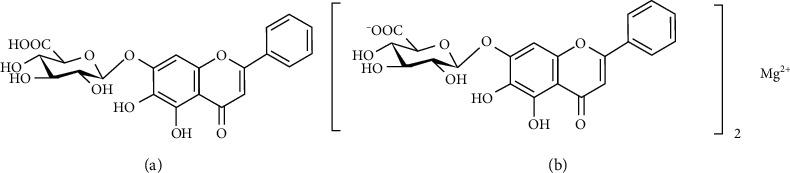
Chemical structures of baicalin (a) and baicalin-Mg (b).

*Scutellaria baicalensis Georgi* (Scutellaria), commonly known as a type of “antipyretic-detoxicate drugs” in traditional Chinese herbal medicine, is widely used to treat “cough with lung heat” [8]. Clinically, a variety of Chinese patent medicine injections containing Scutellaria or its extracts have been widely used for the treatment of respiratory diseases and exerted good efficacy whether used alone or in combination [9–11]. Baicalin (BA, baicalein-7-O-glucuronide flavone, Figure 1(a)) is the major active flavonoid compound isolated from Scutellaria [12]. Although BA has various important biological functions, including antioxidant, anti-inflammatory, and antiviral effects [13, 14], its poor water solubility limits its wide clinical application. Various methods have been used to improve the water solubility of BA; however, the effects are still not very satisfactory. BA is mainly extracted from Scutellaria via water extraction and acid deposition, which is recorded in Chinese Pharmacopoeia [15]. Recently, we isolated a type of BA with good aqueous solubility from Scutellaria without adding acid throughout the process of extraction and purification (the relevant content has been authorized by a US patent [16]), namely, BA magnesium salt (BA-Mg, Figure 1(b)). The water solubility was significantly improved from 0.058 mg/mL (BA) to 22.4 mg/mL (BA-Mg), showing an increase of 300 times.

The lipopolysaccharide- (LPS-) induced model of ALI is widely used in the related mechanism research and drug development [17]. After LPS exposure, toll-like receptor 4 (TLR4), an innate immune receptor of bacterial endotoxins, is recruited to activate the nuclear factor kappa B (NF-*κ*B) signaling transduction pathway and consequent trigger inflammatory response [18, 19]. Numerous studies have indicated that BA exerts its antioxidant and anti-inflammatory functions by inhibiting inflammatory cytokines production via inactivating the NF-*κ*B pathway in LPS-challenged mice [20, 21]. In this study, we found BA-Mg could better prevent and treat ALI triggered by LPS than BA and MgSO_4_. The protection mechanism was probably proposed that BA-Mg would alleviate the inflammatory cascade by inhibiting TLR4-mediated NF-*κ*B signaling pathway that occurred in mice with ALI induced by LPS.

## 2. Materials and Methods

### 2.1. Reagents and Chemicals

BA (CAS registry no. 21967-41-9; molecular formula, C_21_H_18_O_11_; molecular weight, 446.36; batch number 110715-201318) was provided by the National Institute for Food and Drug Control, Beijing, China. Magnesium sulfate was supplied by Jingqiu Chemical Chemical Co., Ltd (Beijing, China). BA-Mg was given by Professor Liu Cuizhe, Institute of Traditional Chinese Medicine, Chengde Medical College. LPS (Escherichia coli serotype 055:B5) was purchased from Sigma-Aldrich (St. Louis, MO, USA). Dexamethasone (DEX) was produced by Aladdin Biochemical Technology Co., Ltd (Shanghai, China). Myeloperoxidase (MPO), malonaldehyde (MDA), and superoxide dismutase (SOD) assay kits were purchased from Nanjing Jiancheng Bioengineering Institute (Nanjing, China). Water used in all experiments was purified by a Milli-Q Biocel Ultrapure Water System (Millipore, Bedford, MA). Enzyme-linked immunosorbent assay (ELISA) kits for tumor necrosis factor- (TNF-) *α*, interleukin- (IL-) 1*β*, and IL-6 were obtained from R&D Systems, Inc. (Minneapolis, MN, USA). Primary antibodies against p-I*κ*B*α*, I*κ*B*α*, MyD88, NF-*κ*B p65, p-NF-*κ*B p65, and TLR4 were obtained from Cell Signaling Technology (Danvers, MA, USA). Dulbecco's modified Eagle's medium (DMEM) was purchased from Life Technologies (Carlsbad, CA, USA).

### 2.2. Animals

Thirty-five male ICR mice (6-8 weeks old, 18-22 g, specific pathogen-free) were purchased from Zhejiang Laboratory Animal Center (License no. SCXK (Zhe) 2019-0002) and were housed in an SPF-barrier facility (22 ± 2°C, 40% to 60% humidity, and 12 h light/dark cycles) in the Laboratory Animal Research Center of Zhejiang Chinese Medical University (certificate number SYXK (Zhe) 2018-0012). All animal care and experimental procedures were abided by the National Institutes of Health Guidelines for the Care and Use of Laboratory Animals (IACUC-20180716-10).

### 2.3. LPS-Induced ALI and Treatment

Mice were randomly divided into 7 groups, 5 in each group: control group, LPS group, LPS + BA − Mg (100, 200 mg/kg), LPS + BA (195.2 mg/kg), LPS + MgSO_4_ (5.2 mg/kg), and LPS + DEX (5 mg/kg). The BA group and MgSO_4_ group were administered with BA and MgSO_4_ equivalent to 200 mg/kg BA-Mg, respectively. Briefly, all mice were pretreated with drugs intraperitoneally once each day for 3 days consecutively. On the fourth day, mice were anesthetized by 2.5% isoflurane inhalation and stimulated with 50 *μ*L LPS (16 mg/mL) via dripping into the nasal cavity. Drugs were intraperitoneally injected 3 days post-LPS exposure. Mice were sacrificed at 1 h after the end of administration on the 6th day; then, bronchoalveolar lavage fluid (BALF) and lung tissues were obtained. All samples are stored at -80°C until assay.

### 2.4. Lung Wet-to-Dry Weight Ratio

The right lung was quickly removed; a filter paper was used to dry the blood on the surface of the lungs, then weighed (wet weight, *W*). Subsequently, the lungs were placed in an oven at 80°C for 48 h to obtain the dry weight (*D*). The lung *W*/*D* ratio was calculated to assess the extent of pulmonary edema.

### 2.5. Histological Examination

The lung tissues were fixed with 10% formalin, followed by dehydration using an alcohol gradient and embedment in paraffin. The 5 *μ*m thick sections were stained with hematoxylin and eosin (H&E) staining for analysis of pathological changes. The degree of pathological injury was scored as described previously [22, 23].

### 2.6. Inflammatory Cell Counts in BALF

The BALF was centrifuged at 3,000 rpm for 10 min at 4°C, and the supernatant was collected for cell quantification. The cell counts were calculated using a cell counter. The number of each cell type was calculated as the total cell number multiplied by the corresponding subgroup percentage.

### 2.7. Inflammatory Cytokine Assay

The levels of TNF-*α*, IL-1*β*, and IL-6 in BALF were analyzed using the commercially available ELISA kits according to the corresponding manufacturer's instructions.

### 2.8. Measurement of Biochemical Indicators

Lung tissues were weighed and homogenized in cold PBS. The MPO, MDA, and SOD were detected using the assay kits according to the corresponding manufacturer's instructions.

### 2.9. Cell Culture

Human lung bronchial epithelial cells (BEAS-2B) were purchased from American Type Culture Collection (Manassas, VA, USA). BEAS-2B cells were cultured in DMEM containing 10% heat-inactivated fetal bovine serum, 100 IU/mL penicillin, and 100 IU/mL streptomycin at 37°C in a humidified atmosphere with 5% CO_2_. The cells were assigned into five groups: control group, LPS group, LPS + BA − Mg (5 *μ*M), LPS + BA (10 *μ*M) group, and LPS + MgSO_4_ (5 *μ*M) group. Briefly, BEAS-2B cells in log-phase were seeded onto 6-well culture plates at a density of 1 × 10^6^ cells/mL for 24 h. Cells were pretreated with BA-Mg, BA, and MgSO_4_ 6 h before LPS stimulation (8 *μ*g/mL). The levels of IL-6, IL-1*β*, and TNF-*α* of supernatants were detected with ELISA kits according to the manufacturers. And the related protein expressions of TLR4/NF-*κ*B were measured by western blot assay.

### 2.10. Western Blot Analysis

Total proteins of lung tissues and BEAS-2B cells were extracted with lysis buffer according to a standard protocol. Afterward, proteins were separated by 10% sodium dodecyl sulfate-polyacrylamide gel electrophoresis, electrotransferred onto a polyvinylidene fluoride membrane, and blocked with 5% skimmed milk at room temperature. Subsequently, membranes were incubated overnight at 4°C with primary antibodies for p-I*κ*B*α*, I*κ*B*α*, NF-*κ*B p65, p-NF-*κ*B p65, MyD88, and TLR4, respectively, and subsequently incubated with horseradish peroxidase- (HRP-) conjugated secondary antibodies (1 : 5000). Finally, specific protein bands were visualized and quantified using the ECL (Millipore, USA).

### 2.11. Statistical Analysis

The GraphPad Prism 8.0 software (GraphPad Software, La Jolla, CA, USA) was used for all statistical analyses. Data were analyzed by one-way analysis of variance (ANOVA) followed by Tukey's test. A *P* value of <0.05 was considered statistically significant. All data are expressed as mean ± standard deviations (SD).

## 3. Results

### 3.1. BA-Mg Alleviated LPS-Induced ALI in Mice

**Figure 2:**
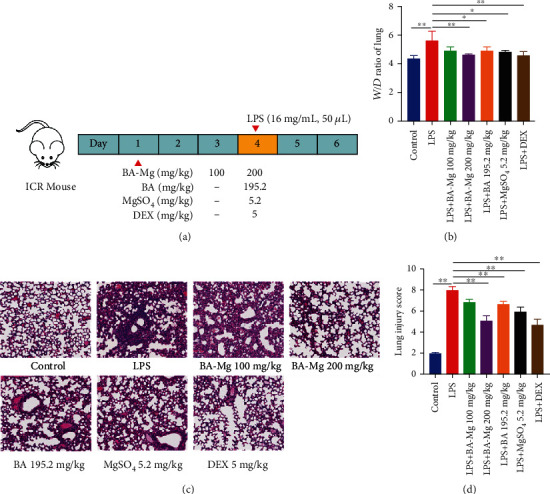
BA-Mg alleviated LPS-induced ALI in mice. (a) Experiment design. Mice were injected intraperitoneally BA-Mg, BA, and MgSO_4_ for 3 days before nasal instillation of LPS, and samples were collected at 48 h after LPS stimulation. (b) Lung *W*/*D* ratio. (c) Representative H&E staining of lung tissue sections. (d) Histological score. LPS: lipopolysaccharide; BA-Mg: baicalin-Mg; BA: baicalin; DEX: dexamethasone. Data were represented as mean ± SD (*n* = 5). ^∗^*P* < 0.05, ^∗∗^*P* < 0.01 versus LPS group.

We evaluated the protective efficacy of BA-Mg, BA, and MgSO_4_ in the LPS-induced ALI model (Figure 2(a)). Pulmonary edema is the hallmark of ALI; the *W*/*D* ratio is often used to evaluate the extent of pulmonary edema. Compared with the control group, the *W*/*D* ratio was significantly increased than that in the LPS group (*P* < 0.01, Figure 2(b)). Conversely, treatment of BA-Mg, BA, MgSO_4_, and DEX decreased the *W*/*D* ratio compared to the LPS group (*P* < 0.05 or *P* < 0.01), while BA-Mg was more effective than BA and MgSO_4_.

Morphological changes in the lungs were performed with H&E staining, and the degree of lung injury was scored in each mouse, as indicated in Figure 2(c). Herein, there were no obvious histological alterations of lung sections obtained from the control group, characterized by the intact structure and clear pulmonary alveolus. By contrast, mice challenged with LPS exhibited characteristic ALI signs of histopathological damages, evidenced by the severe inflammatory cells diffuse infiltration and destruction of the alveolar histological structure. Consistent with the injury, the total pulmonary injury scores in the LPS group were notably improved compared with that in the control group (*P* < 0.01, Figure 2(d)). However, the above appearance of lesions was pronouncedly mitigated in mice undergoing BA-Mg, BA, MgSO_4_, and DEX administration. Particularly, treatment with BA-Mg at a dose of 200 mg/kg markedly restored the morphology of alveoli, as well as the presence of mild inflammatory cells; moreover, the amelioration was better than that of BA and MgSO_4_. Furthermore, the same trend also appeared in the pathology scoring results (*P* < 0.01, Figure 2(d)). This result demonstrated that pretreatment with BA-Mg could better protect against LPS-induced ALI.

### 3.2. BA-Mg Attenuated LPS-Induced Inflammatory Cell Infiltration and Proinflammatory Cytokine Expression in BALF

**Figure 3:**
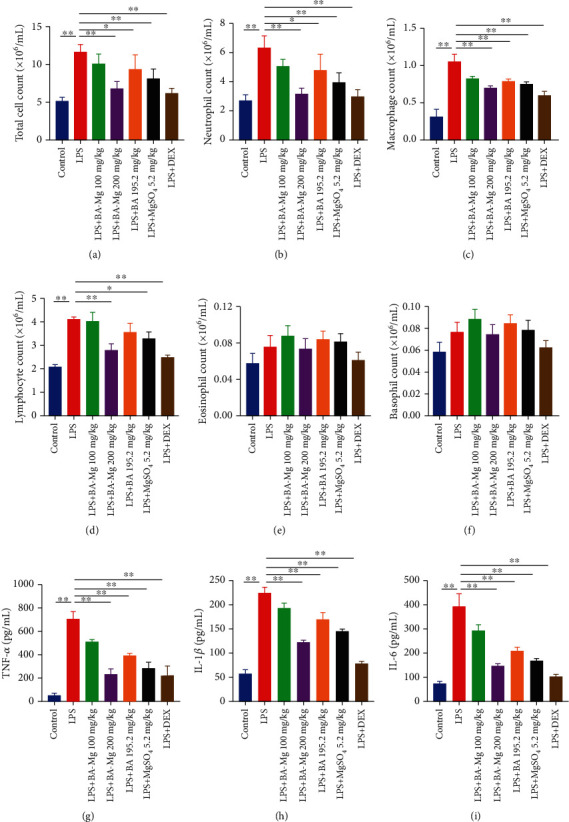
BA-Mg attenuated inflammatory cell infiltration and proinflammatory cytokine expressions in BALF: (a–f) inflammatory cell counts; (g–i) proinflammatory cytokines. Data were represented as mean ± SD (*n* = 5). ^∗^*P* < 0.05, ^∗∗^*P* < 0.01 versus LPS group.

To further investigate the protective effect of BA-Mg, we determined the potential variations of inflammatory cell counts and proinflammatory cytokines in BALF. Firstly, cytological classification and cell counts were performed to evaluate the severity of inflammatory cell infiltration in the lung. We have detected a total of five types of infiltrated immune cells in BALF, including neutrophils, macrophages, lymphocytes, eosinophils, and basophils, as illustrated in Figures 3(b)–3(f). The number of total cells (Figure 3(a)), neutrophils, macrophages, and lymphocytes was significantly different, but there was no significant difference in eosinophil and basophil counts. LPS exposure induced excessive inflammatory cell recruitment compared to that in the control group, as shown by the increased number of total cells, neutrophils, macrophages, and lymphocytes (*P* < 0.01). As expected, BA-Mg, BA, MgSO_4_, and DEX-treated animals could markedly attenuate the numbers of infiltrated inflammatory cells in BLAF compared to the LPS-exposed animals (*P* < 0.05 or *P* < 0.01); but the number of lymphocytes in BALF did not differ significantly in the BA and LPS group. In addition, the effect of BA-Mg was more significant than BA and MgSO_4_.

Additionally, the proinflammatory cytokine levels, such as TNF-*α*, IL-1*β*, and IL-6 in BALF, were assessed by ELISA for further illuminating the anti-inflammatory effect of BA-Mg. In comparison with the control group, the concentrations of TNF-*α*, IL-1*β*, and IL-6 in the LPS group were significantly increased (*P* < 0.01), whereas BA-Mg, BA, MgSO_4_, and DEX pronouncedly attenuated this increasing trend (*P* < 0.05 or *P* < 0.01, Figures 3(g)–3(i)). Notably, pretreatment with BA-Mg was more effective than an equimolar dose of BA and MgSO_4_ in inhibiting the expressions of cytokines and chemokines. Collectively, these findings might indicate that BA-Mg could inhibit inflammatory cell gathering and downregulate relevant proinflammatory factors in LPS-induced inflammatory response.

### 3.3. BA-Mg Reduced MPO Activity in Lung Tissue

**Figure 4:**
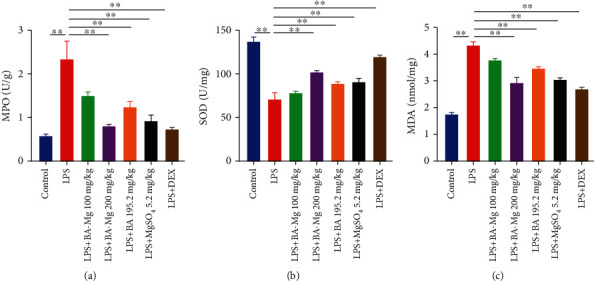
Effect of BA-Mg on the MPO (a), MDA (b), and SOD (c) levels in lung tissue of mice injured by LPS. Data were represented as mean ± SD (*n* = 5). ^∗^*P* < 0.05, ^∗∗^*P* < 0.01 versus LPS group.

It was observed that MPO activity, as a specific marker of inflammatory cell extravasation, was significantly upregulated in LPS-induced mice as compared to the control tissues (*P* < 0.01, Figure 4(a)). Of note, administration of BA-Mg, BA, MgSO_4_, and DEX efficiently reversed this change in the LPS group (*P* < 0.01), whereas the effect of BA-Mg was more significant.

### 3.4. Protective Effects of BA-Mg against Oxidative Stress in ALI Mice

The oxidative stress might result in alveolar epithelial cell apoptosis and lung destruction. Thus, the levels of SOD and MDA were investigated to assess the oxidative status, as indicated in Figures 4(b) and 4(c). After inhalation of LPS, SOD activity was dramatically lower (*P* < 0.01), and MDA levels were sharply higher (*P* < 0.01) with respective to the control group. The pretreatment with BA-Mg, BA, MgSO_4_, and DEX pronouncedly increased SOD levels and decreased MDA content (*P* < 0.01) compared with the LPS group. Moreover, in terms of ameliorating the SOD activity and MDA content, BA-Mg was more effective than BA and MgSO_4_. Overall, these results indicated that BA-Mg pretreatment significantly suppressed oxidative stress by increasing antioxidant enzyme activity in LPS-induced ALI.

### 3.5. BA-Mg Inhibited Activated TLR4/NF-*κ*B Signaling Pathway in LPS-Stimulated ALI Mice

**Figure 5:**
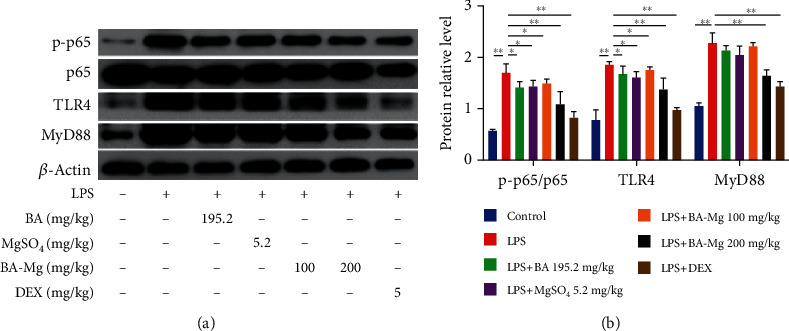
Effect of BA-Mg on TLR4/NF-*κ*B signaling pathway in lung tissue of mice injured by LPS: (a) western blot analysis; (b) statistical results of western blot analysis. Data were represented as mean ± SD (*n* = 5). ^∗^*P* < 0.05, ^∗∗^*P* < 0.01 versus LPS group.

To investigate whether BA-Mg exerted anti-inflammatory effects responded to the LPS challenge by regulating the TLR4/NF-*κ*B signaling pathway, we tested the protein expression of TLR4/NF-*κ*B signaling pathway by western blotting, as illustrated in Figure 5. In the LPS group, the expressions of p-p65, TLR4, and MyD88 protein were upregulated (*P* < 0.01), which were reversed by pretreating with BA-Mg, BA, and MgSO_4_ (*P* < 0.05 or <0.01), especially in the BA-Mg high-dose group (*P* < 0.01). This finding suggested that BA-Mg could suppress the TLR4/NF-*κ*B signaling pathway to relieve inflammatory response of ALI.

### 3.6. Effect of BA-Mg on LPS-Induced BEAS-2B Cells In Vitro

**Figure 6:**
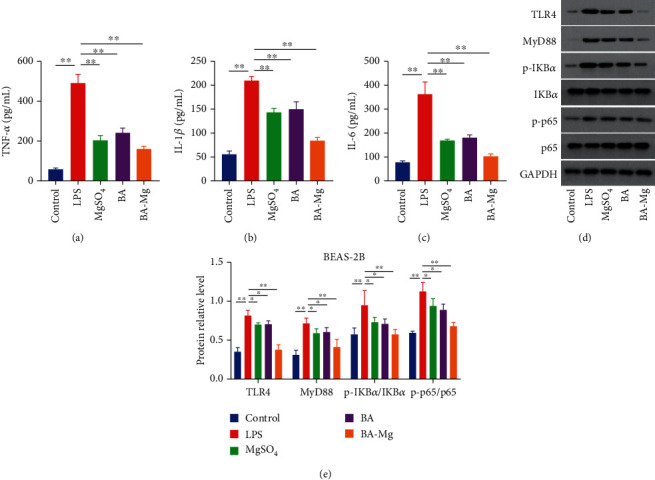
Effect of BA-Mg on LPS-induced BEAS-2B cells in vitro. (a–c) Effect of BA-Mg on inflammatory cytokine expressions in LPS-induced BEAS-2B cells. (d, e) Effect of BA-Mg on TLR4/NF-*κ*B signaling pathway in LPS-induced BEAS-2B cells. At least 3 independent repetitions per experiment were conducted. ^∗^*P* < 0.05, ^∗∗^*P* < 0.01 versus LPS group.

To further confirm the anti-inflammatory mechanism of BA-Mg, the inflammatory cytokine expressions of TNF-*α*, IL-1*β*, and IL-6 were measured in LPS-stimulated BEAS-2B cells in vitro. As revealed in Figures 6(a)–6(c), consistent with the results in vivo, LPS exposure significantly increased the expressions of TNF-*α*, IL-1*β*, and IL-6 (*P* < 0.01), whereas this increasing trend was pronouncedly attenuated by BA-Mg, BA, and MgSO_4_ (*P* < 0.01). Collectively, these findings might indicate that BA-Mg could inhibit inflammatory cell gathering and downregulate relevant proinflammatory factors in LPS-induced inflammatory response.

Moreover, the protein expressions of TLR4, p-p65, p-I*κ*B, and MyD88 were measured in LPS-stimulated BEAS-2B cells in vitro. As showed in Figures 6(d) and 6(e), consistent with the results in vivo, LPS exposure significantly upregulated the expressions of TLR4, p-p65, p-I*κ*B, and MyD88 compared with those in the control group (*P* < 0.01). As expected, pretreatment with BA-Mg, BA, and MgSO_4_ markedly downregulated the levels of TLR4/NF-*κ*B signaling pathway compared with the LPS group (*P* < 0.05 or *P* < 0.01). Furthermore, pretreatment with BA-Mg exhibited more efficient inhibitory effects than BA or MgSO_4_. The results further implied that BA-Mg exhibited anti-inflammatory activity via regulating TLR4/NF-*κ*B signaling pathway.

## 4. Discussion

BA is the main active ingredient of Scutellaria, and its content is generally 9%-20%. There is abundant evidence suggesting that BA exerts clear anti-inflammatory effects on a variety of inflammation models and has protective and therapeutic effects on ALI [24, 25]. However, BA has poor aqueous solubility, which sharply decreases its bioavailability. Previously, we reexamined the extraction process of BA and obtained a good water-soluble BA-Mg. In the present study, we provided scientific supportive evidence that BA-Mg could pronouncedly ameliorate LPS-induced ALI than BA.

The destruction of the alveolar-capillary barrier and inflammatory disorder is the typical pathophysiological characteristics response to ALI induced by LPS exposure [26–28]. In this study, we found that BA-Mg pretreatment efficiently reduced the *W*/*D* ratio in comparison to the LPS group, indicating that BA-Mg could reduce lung edema by protecting the capillary-alveolar barrier. Meanwhile, BA-Mg dramatically reduced the infiltration of total cells, neutrophils, macrophages, and lymphocytes in BALF and suppressing MPO activity in lung tissues of LPS-induced ALI mice. The MPO activity, a prooxidant enzyme, and direct biomarker of neutrophil accumulation reflected the pulmonary inflammation and cell damage in ALI [3]. In the histopathological analysis, we observed the attenuated histopathological changes after BA-Mg administration, such as lung edema, inflammatory cell infiltration, and thickening of the alveolar wall, were manifestly different from the LPS group. Furthermore, the balance of oxidative defense is also crucial for the therapy of ALI [20]. BA-Mg could improve SOD activities and reduce MDA content, the generally accepted indicators for evaluating the extent of lipid peroxidation. These findings illustrated that BA-Mg protected against LPS-induced ALI by enhancing antioxidant systems and attenuating the inflammatory cell infiltration in the lungs.

**Figure 7:**
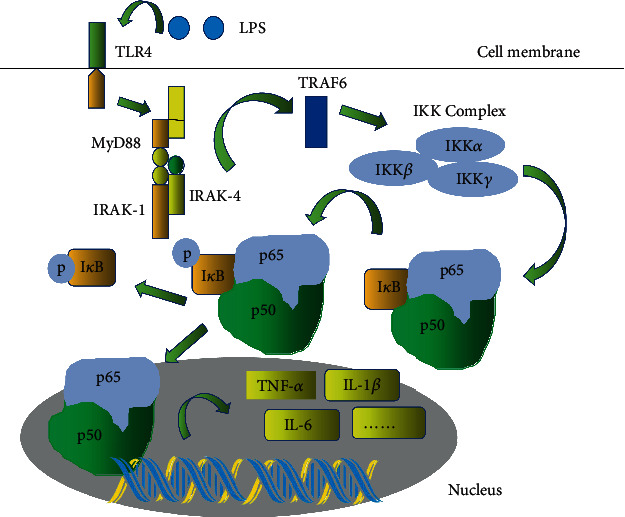
Regulation of LPS-induced TLR4/NF-*κ*B signaling pathway by ubiquitination and phosphorylation. When NF-*κ*B is not subjected to external stimulation, it can induce the assembly of an inactive I*κ*B/p50/p65 trimer. After LPS stimulation, the activated TLR4 firstly forms a complex with MyD88. Then, the MyD88 complex promotes the phosphorylation of IRAK4, thereby further phosphorylates TRAF6, while detaching from MyD88. Ubiquitination of TRAF6 induces downstream IKK-mediated NF-*κ*B signaling pathway, triggering downstream I*κ*B phosphorylation and inactivation, which leads to NF-*κ*B dimer breaking away from I*κ*B, thereby entering the nucleus and acting on the original NF-*κ*B response element, that is, the gene locus of binding to NF-*κ*B. Thereafter, the transcriptional translation process is initiated, which leads to the expression of downstream genes related to inflammatory factors and chemokines.

Consistent with the inhibition of inflammatory cell infiltration, BA-Mg remarkably downregulated the overproduction of TNF-*α*, IL-1*β*, and IL-6 in BALF of LPS-treated mice. It is well-known that TLR4 is a key functional protein that responds to LPS [29]. Upon LPS stimulation, TLR4 activation leads to the phosphorylation of NF-*κ*B p65, stimulating the production of inflammatory factors such as TNF-*α*, IL-1*β*, and IL-6, which could recruit inflammatory cells to initiate immune-inflammatory responses in ALI (Figure 7) [18, 30]. These inductions ultimately caused the infiltration of inflammatory cells into severe lesions [29, 31]. Thus, blocking TLR4/NF-*κ*B signaling pathway could mitigate inflammatory response triggered by LPS [32–34]. After the LPS challenge, the protein expression of the TLR4/NF-*κ*B signaling pathway in lung tissues as well as in BEAS-2B cells was elevated. It is worth noting that BA-Mg markedly suppressed the activation of the TLR4/NF-*κ*B signaling pathway, thereby ameliorating ALI in mice.

Although the protective effect of BA and MgSO4 in ALI under LPS exposure, the effect of BA-Mg was significantly better than that of BA and MgSO_4_ in ameliorating symptoms of ALI. As we all know, the Mg ion is essential for maintaining health, as it is involved in almost all cell functions, including phosphorylations, enzymatic processes, energy balance, protein synthesis, and DNA stability [35]. According to the documented reports, dysregulated Mg homeostasis seems to be the basis of the pathophysiology of diverse diseases [36]. In particular, Mg deficiency has been closely related to the development of various inflammation-driven diseases [37, 38]. It is suggested that supplementing Mg, as an adjuvant, may be beneficial to treat inflammation [37]. Previously, we had demonstrated that the ameliorative effect of BA-Mg pretreatment on CCl_4_-induced acute liver injury in mice through regulating inflammatory cytokine production and antioxidant stress [39]; moreover, the pharmacological activity of BA-Mg was better than BA and MgSO_4_ [40].

## 5. Conclusion

In conclusion, we have demonstrated that BA-Mg, a good water-soluble compound extracted from Scutellaria, protected against LPS-induced ALI in mice. Mechanistically, it was probably attributable to the suppression of inflammation via downregulating TLR4/NF-*κ*B signaling pathway and inhibiting oxidative stress. However, the optimal prevention time point and frequency of administration of BA-Mg will be further verified in future experiments. In any case, BA-Mg, as a new drug structure, is a promising therapeutic candidate drug for the treatment of ALI.

## Data Availability

The (data type) data used to support the findings of this study are included within the article.
